# Supplementary material for: Seasonal regimes of warm Circumpolar Deep Water intrusion toward Antarctic ice shelves
Source: Commun Earth Environ. 2025 Mar 1;6(1):168. doi: 10.1038/s43247-025-02091-w (PMC11872733; doi:10.1038/s43247-025-02091-w)
Supplement: Supplementary file 2 — Supplementary Information [file 43247_2025_2091_MOESM2_ESM.pdf]

Supplementary Information

J. Lanham[1], M. Mazloff[2], A. C. Naveira Garabato[3], M. Siegert[4], and A. Mashayek[1]

[1]Department of Earth Sciences, University of Cambridge, UK
[2]Scripps Institution of Oceanography, University of California, USA
[3]National Oceanography Centre, University of Southampton, UK
[4]University of Exeter, UK

May 2024

S1 Supplementary Methods

S1.1 Supplementary Method: ASF Detection

We devise a metric to detect the ASF in SOHI, which we use in the introductory Figure 1 and also to differentiate between the wind-driven ASF and undercurrent regimes. This is defined using the meridional derivative of sea surface height (SSH), given that we expect elevated SSH on the shelf when the ASC is strong, due to depth-integrated shoreward Ekman transport. The ASF conditions are satisfied when the following are true across a meridional spacing of $n = 30$ grid boxes:

$$\frac{dSSH}{dY} > 0, u < -0.05 \text{ m s}^{-1}, \quad (1)$$

We also impose a depth constraint 800 m $< Z_{bottom} <$ 2000m to isolate the near-shelf ASC flow, and exclude the influence of the Antarctic Circumpolar Current further equatorward. A 1000 grid point rolling average of the ASF is shown against longitude in Figure S2.

S1.2 Supplementary Method: End Member Selection

We classify water masses in our model using a least-squares fit to end members, which is similar to classical Optimum Multi-Parameter (OMP) analysis [1]. OMP analysis uses a series of linear mixing equations in order to establish a relative water mass contribution for a set of known (or in this case modelled) ocean variables. Values of these variables are selected to represent characteristic unmixed 'Source Water Types' (SWTs); i.e. the properties that are typical of a water mass at the point of formation. From this, known variable values at each point in space can be considered to be a linear combination of each of the SWTs, such that it is possible to determine the spatial distribution of each SWT.

We define end members for 4 STWs: Circumpolar Deep Water (CDW), Dense Shelf Water (DSW), Low Salinity Shelf Water (LSSW) and Ice Shelf Water (ISW). End members are picked from the extremes in potential temperature (θ)-Salinity (S) parameter space shown in Figure S18. CDW is chosen as the warm, salty tail of the distribution (Blue x in Figure S18). The final values of $\theta = 2$°C and $S = 34.7$ are within the observed CDW parameter space [2]. DSW is chosen as the cold, salty tail of the distribution (orange x in Figure S18). The monthly decomposition reveals a lack of DSW in austral summer, consistent with knowledge that DSW is formed in austral winter. The end member values of $\theta = -1.9°C$ and $S = 34.7$ are within the observed parameter space of DSW [3]. The LSSW end member is defined as $\theta = -1.7°C$ and $S = 34.3$. These are selected as the relative midpoint within the narrow observed T-S range of LSSW [2]. ISW is selected as the

cold, fresh tail of the distribution ($\theta = -1.9°C$ and $S = 33.6$). In reality, we acknowledge that this end member likely encompasses both ISW in the model's parameter space and also cold-capped fresh Winter Water. This is discussed in more detail in Section S9.1.

S1.3 Supplementary Method: Water Mass Classification: Machinery

We select values of θ and S to represent four unmixed SWTs as outlined in Section S1.2. We can write the resulting set of 3 linear mixing equations (2 for θ and S, and 1 for mass conservation):

$$\sum_{i=1}^{n} x_i * \theta_i^{SWT} = \theta^{Model} + r_\theta \tag{2}$$

$$\sum_{i=1}^{n} x_i * S_i^{SWT} = S^{Model} + r_S \tag{3}$$

$$\sum_{i=1}^{n} x_i = 1 + r_{Mass} \tag{4}$$

Each variable is normalised by the mean and standard deviation, such that:

$$\theta^{norm} = (\theta - \bar{\theta})/\theta_\sigma \tag{5}$$

$$S^{norm} = (S - \bar{S})/S_\sigma \tag{6}$$

where the standard deviation of each variable, t_σ, is given by: $t_\sigma = \sqrt{\sum(t_i - \bar{t})^2/n}$. Equations (2)-(4) are solved simultaneously via a least squares method to give a vector $\mathbf{x}$ that minimises the residuals r_θ, r_S and r_{Mass}. We use the machinery from Wunsch (2005), starting by defining a water mass matrix $\mathbf{H}$ to describe the four SWTs that we specify in Section S1.2:

$$\mathbf{H} = \begin{bmatrix} \theta_{CDW}^{SWT} & S_{CDW}^{SWT} & 1 \\ \theta_{LSSW}^{SWT} & S_{LSSW}^{SWT} & 1 \\ \theta_{DSW}^{SWT} & S_{DSW}^{SWT} & 1 \\ \theta_{ISW}^{SWT} & S_{ISW}^{SWT} & 1 \end{bmatrix}$$

We define a diagonal matrix, $\mathbf{R}$, to describe the error variance v on each of the variables:

$$\mathbf{R} = \begin{bmatrix} v_\theta & 0 & 0 \\ 0 & v_s & 0 \\ 0 & 0 & v_{Mass} \end{bmatrix}$$

In practice, values of v determine the strength of the constraint on each variable: i.e. the extent to which residuals in that variable will be tolerated in the solving of the Equations (2)-(4). In this study, we choose to impose a harder constraint on mass ($v_{\theta,S} = 0.01, v_{Mass} = 0.0001$) to adhere more strongly to mass conservation.

We define another diagonal matrix, $\mathbf{P}$, which contains the error variance v on each of the water masses:

$$\mathbf{P} = \begin{bmatrix} v_{CDW} & 0 & 0 & 0 \\ 0 & v_{LSSW} & 0 & 0 \\ 0 & 0 & v_{DSW} & 0 \\ 0 & 0 & 0 & v_{ISW} \end{bmatrix}$$

$\mathbf{P}$ can be thought of as an *a priori* likelihood relating to each water mass. We choose to assume an equal weighting in this study: $v_{SWT} = 1/n^2$, where n is the number of STWs (in this case, $n = 4$).

Finally, for every grid point in the model, we define a vector $\mathbf{y}$, which contains the modelled variables and a mass term:

$$\mathbf{y} = \begin{bmatrix} \theta^{norm} & S^{norm} & 1 \end{bmatrix}$$

The matrices are then solved to give the solution vector $\mathbf{x}$, which contains the least squares fit to Equations (2)-(4) (i.e. the water mass fraction for each of the four SWTs):

$$\mathbf{x}_{gridcell} = (\mathbf{H}^T\mathbf{R}^{-1}\mathbf{H} + \mathbf{P}^{-1})^{-1}\mathbf{H}^T\mathbf{R}^{-1}\mathbf{y} \tag{7}$$

where $\mathbf{H}^T$ denotes the transposed water mass matrix $\mathbf{H}$. The residuals of the solution can be expressed by the vector $\mathbf{e}$:

$$\mathbf{e}_{gridcell} = \mathbf{y} - \mathbf{H}\mathbf{x}_{gridcell} \tag{8}$$

This set of equations is solved for every grid point in SOHI, which, when returned to the SOHI grid, yields four fully-gridded water mass estimates for each of the SWTs defined in Section S1.2. Unlike most OMP methods, we intentionally do not include a non-negative constraint in our least squares equations. This is to reduce the risk of over-fitting; rather than force the solution of the analysis to be positive, we prefer to use the presence of significant negatives to identify regions where modelled values are out of the range of the selected end members, and thus where our prescribed error variance $\mathbf{R}$ is underestimated. Importantly, the regions where significant negatives occur are away from the shelf, where our analysis is necessarily less constrained (see Section S8.1).

S1.4 Supplementary Method: Significance Testing of Correlations

We test the ERA5 zonal wind stress - CDW correlations in Figure 4b for significance at the 90, 95 and 99th percentile. These are shown in Figure S4. The thresholds are taken from the Pearson correlation coefficient critical value table, with 10 degrees of freedom ($N = 12, df = N - 2$). R values are significant at the 99% level in some parts of the shelf between 30-60°E, in the western Amundsen Sea and also in Vincennes Bay to the west of the Browning Peninsula. At the 95% level, the significance in this area expands to include the Totten Glacier (100-120°E). The vast majority of the negative correlations shown in Figure 4b are not significant at the 95% level. The Antarctic Peninsula shows some statistically significant negative r-values, which may be due to strong eddy activity. This is discussed in Section S3.

We check the canyon correlations in Section 2.2 for significance by the same method. Both the correlations in the Weddell and Ross seas are significant at the 99% level. Most of the undercurrent correlation is found to be significant at the 99% level, which is shown as stippling in Figure 7.

S2 Supplementary Note: Advection near the Fimbulisen Ice Shelf

The shelf region in the far eastern Weddell Sea near and to the west of the Fimbulisen Ice Shelf shows significant levels of seasonal variance, but is anomalous to the regimes set out in the main discussion. It appears to be negatively correlated to wind forcing but, unlike the Adélie Coast, does not have sufficient buoyancy forcing to pace the cycle of seasonal DSW intrusion. Here we suggest that the seasonality of CDW on the shelf is primarily the result of CDW advection from the east.

In Section 2.1, it was shown that the region directly eastward of this shelf area near the Lazarev shelf (i.e. upstream considering that the mean direction of near-shelf ASC flow is westward) experiences substantial summer CDW intrusion, forced by a wind-induced relaxation of the ASF. Given that westward velocities are particularly high here (shown in Figure S3a), we suggest that the upstream CDW that moves shelfward in summer is advected westward in the following months. This would account for the apparent negative correlation with zonal wind. Assuming that the seasonal wind forcing upstream and downstream are broadly in line (which is indeed suggested by

Section S4), advection of CDW into the Fimbulisen shelf begins to increase as the direction of wind forcing switches to become more easterly. Figure S3b plots the month of maximum CDW at the sea floor, filtered by the seasonal variance criterion set out in the main text. Upstream, near and to the east of the Lazarev shelf, CDW peaks in summer in accordance with the timing of the westerly wind maximum. From this point westward, the month of maximum CDW gets progressively later as a function of decreasing longitude, reaching August at 10°W. This suggests an advective timescale of approximately ∼8 months per 60°longitude, or 7.5 °longitude per month. This is generally consistent with the results of Dawson *et al.* [4], who find that the advective timescale within the 33°W - 72°E is 1.6 years, which equates to 5.5 °longitude per month. The discrepancy can likely be accounted for by the fact that our section is within the zonal flow maximum in Figure S3a.

S3 Supplementary Note: The Antarctic Peninsula

There is a section on the northern-western side of the Antarctic Peninsula that shows significant seasonal variance in Figure 3b. The correlation with ERA5 zonal wind time series is negative and statistically significant at the 95% level, shown in Figure S4. However, we do not classify this region in either of our wind or buoyancy-driven regimes. This is because observational evidence in this area suggests that there is not a pronounced and systematic seasonal signal in on-shelf water mass properties [5]. Firstly, there is very little buoyancy forcing in the region: DSW is not produced, and the peninsula largely isolates West Antarctica from the downstream advection of DSW [4]. If there was a wind-driven seasonal cycle in CDW upwelling (via Ekman suction) in this area, we would expect a positive correlation with zonal winds. Instead, it appears that the dominant mechanism of CDW supply to the western Antarctic Peninsula shelf is eddies shed by the ACC and steered by bathymetry [6][7][5]. Therefore, we suggest that the seasonal variance of CDW concentration that is shown throughout the season along this section of the shelf stems primarily from aliased eddy variability, rather than reflecting a systematic seasonal signal related to either wind or buoyancy forcing.

S4 Supplementary Note: ERA5 winds: 2005/06 vs. the Climatological Mean

Given the significance of the wind forcing to the conclusions of this study, it is worth assessing the extent to which the 2005/06 winds were typical of the mean seasonal conditions around Antarctica. Seasonal zonal wind anomalies (summer 3-month average minus winter 3-month average) are calculated for both 2005/06 and a 52-year climatology (1970-2022) and shown in Figures S5a and b. Figure S5a displays a high degree of similarity to the climatology in Figure S5b in both spatial configuration and magnitude of anomaly. Figure S5c shows the difference between each of the seasonal anomalies. Summer wind forcing is slightly more westerly in 2005/6 than the mean over the far eastern Ross Sea, and also along much of the East Antarctic coast between ∼0-120°E. Summer winds are more easterly than usual over the Weddell Sea, the Amundsen and Bellingshausen seas, and the Adélie Coast. In fact, it appears as though the spatial pattern in Figure S5c is similar to the general pattern of seasonal forcing observable in Figures S5a-b. This suggests that the year spanning 2005/06 saw largely an exacerbation in the magnitude of time-mean wind forcing in an absolute sense, rather than any significant interannual variability in the spatial organisation of atmospheric phenomena. This is important for the extrapolation of our results beyond 2005/06. Whilst it is the case that our analysis might overestimate the magnitude of wind-driven seasonal CDW-related processes on the shelf relative to an average year, it is unlikely that we misclassify the dominant mechanisms due to an anomalous spatial pattern of wind forcing.

S5 Supplementary Note: 3-D Topography

To support the discussion provided in Section 2.2, we show 3-dimensional plots of Prydz Bay and the Adélie Coast sections from three angles in Figure S8. The Adélie Coast exhibits a lower mean shelf depth than Prydz Bay, which explains why CDW shows seasonal variance on the Adélie Coast but not Prydz Bay. The geometry of the Adélie shelf is also significant in that it slopes downward poleward of the shelf break, in a configuration associated with Marine Ice Sheet Instability. Thus, once CDW breaches the ASF or shelf break, it flows downwards to fill the depression. This is visible in Figure 8.

S6 Supplementary Note: Assessing the Impact of Model Drift

Despite the fact that the kinetic energy has largely stabilised in SOHI, we acknowledge that there may be some regions on the shelf where θ and S are yet to fully equilibrate. In this section we review the evidence for model drift in both wind and buoyancy driven regimes.

S6.1 Supplementary Note: Wind-Driven Regimes

Figure S6 shows a time series of CDW at three locations in West and East Antarctica (regime 1 is split into 0-60°E and 80-120 °E). Figure S6a shows that the region between 0-60°E shows a systematic seasonal cycle in CDW on the shelf and therefore is unlikely to be largely impacted by model drift. However, both the region between 80-120°E and the west Antarctic margin show significant linear trends across the season, shown in Figures S6d and e.

This could be explained by model drift. However, it is also the case that these regions show significant linear trends in the surface wind forcing during the same period. This is shown in Figures S6f and g. In the section between 80-120°E, there is a linear negative wind trend which accompanies the decline in CDW fractions on the shelf. By the mechanism outlined in Section 2.1, a negative trend in zonal wind velocity over the year could induce a sustained steepening in isopycnals and reduced CDW concentrations on the shelf. Likewise, in Regime 2 / West Antarctica, the sustained positive linear trend in zonal wind velocity could, by the mechanism outlined in Section 2.1, induce the observed trend in CDW fractions on the West Antarctic shelf.

In Figure S7 we show the correlation between the surface zonal wind time series and the de-trended CDW time series. Most of the correlations in the sector in the region 20-60°E remain significant, consistent with the lack of trend in Figure 6a. However, the significance of many of the correlations in the region 80-120°E are lost, suggesting that it was the positive trend in both the CDW fraction and wind velocity (Figures S6d and f) that accounted for a significant part of the correlation. In West Antarctica, the de-trended correlations remain significant despite the trends in Figures S6e and g), suggesting that the winds may drive both the variability and trend across the year.

It is difficult to entangle the relative impacts of drift and longer term variability / trends. This is a natural downside to the short model run time. However, we contend that this is needed in order to reach the resolution necessary to resolve more processes than typical longer-run ocean models are capable of.

S6.2 Supplementary Note: Regime 3: Adélie Coast Drift

As suggested in Section 2.2, we find that the seasonal cycle of both CDW and DSW on the Adélie Coast shelf does not close, and instead shows a near linear trend. In Figure S10a, we show the CDW time series from Figure 8, with the addition of the extra 6-months of model data available. In this case, it appears that the trend stabilises after approximately 1-year, suggesting that the first year of data analysed may be subject to drift.

In order to ensure that the variability in CDW fraction that we note on the Adélie Coast is not wholly due to model drift, we make use of the extra 6-months of model data and re-calculate Figure 3b with a de-trended 18-month CDW time series, shown in Figure S9a. Importantly, the Adélie shelf continues to show significant levels of CDW variance (Figure S9b), when compared with other DSW-producing regions (Ross and Weddell Seas, Prydz Bay in Figure S9c).

In Figure S10b and c, we show the de-trended 18-month CDW and DSW time series for the Adélie Coast and Prydz Bay, respectively. Consistent with the discussion in Section 2.2, both regions have a sufficiently narrow shelf to sustain seasonal variability in DSW formation and export: de-trended DSW fractions vary significantly across the 18-month period in both regions. For the Adélie Coast, the de-trended variability of DSW is almost entirely countered by equal and opposite de-trended CDW variability (Figure S10b). In contrast, in Prydz Bay, the de-trended DSW variability is countered mainly variability in ISW, with CDW undergoing little change (Figure S10c). This is also illustrated by the contrasting CDW variance levels between the two regions in Figures S9b and c, and is consistent with the conclusions made in Section 2.2 relating to the relative depth of the continental shelf (i.e. the Prydz shelf is too shallow to sustain significant CDW intrusion when DSW is exported).

Whilst we acknowledge that there is likely some degree of model drift at the Adélie Coast, we contend that Figures S9 and S10 demonstrate that the main conclusions of Section 2.2 remain unchanged. With all trends removed, both the Adélie Coast and Prydz Bay exhibit substantial variability in DSW fractions on the continental shelf, in contrast with the Ross and Weddell Seas. Moreover, the Adélie Coast remains the only DSW-producing region to exhibit substantial variability in (de-trended) CDW concentrations (Figure S9), and we show in Figure S10 that this is likely related to the production and export of DSW on seasonal timescales.

S7 Supplementary Note: LSSW and ISW Seasonality

We show seasonal variance plots for LSSW and ISW in Figure S11, to accompany the CDW and DSW seasonal variance plots in Figures 3b and d. Figure 3d shows that the narrow shelves that produce DSW (i.e. Adélie Coast and Prydz Bay) sustain a seasonal cycle of DSW production and export and therefore exhibit high seasonal variance values. Both the Weddell and Ross Seas show low DSW seasonal variance, which we attribute in the main discussion to a reservoir effect. The main discrepancy from the CDW seasonal variance plot set out in the main results section is that Prydz Bay shows significant DSW seasonality. Here, the shelf is so shallow that, when DSW is exported, CDW is largely below the level of the shelf break. Instead, LSSW and/or ISW/WW is imported to the shelf (likely via wind-driven Ekman dynamics), evidenced by the significant seasonal variance in Prydz Bay in both Figures S11a and b. In fact, analysis of the extended 18-month time series in Section S6 reveals that it is likely ISW (or WW) that compensates DSW here. LSSW and ISW seasonal variance values are generally high along the East Antarctic coast, which is identified in the main discussion as hosting the ASF-driven regime. This is consistent with the ASF mechanism; as easterly winds strengthen, shore-ward Ekman transport causes fresh, cold surface waters to downwell onto the shelf, forming LSSW. As the slope front relaxes with westerly wind anomalies, CDW is fluxed toward the shelf and LSSW/ISW is exported.

S8 Supplementary Note: Additional SOHI verification with MEOP data

We validate SOHI output on and near the shelf with CTD measurements taken from marine mammals as part of the MEOP consortium [8]. In Figure 2, we show contoured bivariate T-S histograms for a number of near-shelf regions with a high data density of MEOP measurements. These regions are defined as in Narayanan *et al.* [9]. MEOP equivalent measurements are sub-sampled from the model as the nearest grid-point at the nearest date and time, as described in Section 4.2.

The mean and standard error of the MEOP data at each shelf are shown in Table S9.1. The accuracy of MEOP measurements is reported to be ±0.02 °C for temperature and ±0.03 psu for salinity [10]. The standard error of all means in Table S9.1 is within this range, suggesting that all shelves are well-constrained given the measurement uncertainty. We choose to use all MEOP data from the period 2004 to 2017, as in Narayanan *et al.* [9]. Whilst this prevents direct year-to-year comparison and neglects the impact of interannual variability, it is necessary to reduce the risk of under-sampling. We find that MEOP data near the shelf generally do not converge and stabilise until around 5000 data points are considered. A sample of this analysis is shown in Figure S12, which displays the running mean of the sequential addition of a random data point for temperature (red) and salinity (blue) across six shelves. Figure S12f shows the number of MEOP data points for each shelf region displayed in Figure 2 from this period. With the exception of the Leopold and Astrid Coast, in which temperature has not stabilised in Figure S12d and the standard error is an order of magnitude higher than in all other shelves, the remaining shelves have a sample size greater than 5000 and exhibit equilibrium in both temperature and salinity when all data points are used in the running mean.

We show that the two regions with the worst agreement are improved substantially by a depth-decomposition below 400 m, illustrated in Figures S14a and b. As discussed in Section 4.2, we therefore attribute a significant amount of the discrepancy in these regions in Figure 2 to mixed layer properties, which are of little consequence to our study. Figure S14c shows MEOP data density in our sample below 400 m. Only the Bellingshausen and Amundsen seas exceed 3000 data points in this depth range, which means that extending this analysis beyond these two shelves is likely to run the risk of under-sampling. Standard error values below 400 m rise considerably, such that the temperature standard error exceeds the measurement uncertainty; the mean average temperature and salinity standard error across the most sparsely sampled 7 shelves below 400 m are 0.036 °C and 0.016 psu, respectively.

Given the focus of our study, we also compare the seasonality of the model with that in MEOP measurements. Figures S15a-b show the linear regression between modelled and observed monthly temperature and salinity values, respectively. Each data point represents the monthly mean on a given shelf. However, we find that under-sampling limits skill. For example, selecting only the shelves that have monthly samples of over 20,000 increases the Pearson r-value of salinity by 0.25 (Figure S15d). Plotting the minimum monthly sample size against the r-value shows that both temperature and salinity converge to near 1 at ∼35,000 data points. Figure S15e shows a seasonality comparison from the Bellingshausen Sea, a region that is particularly well sampled in the MEOP dataset.

S8.1 Supplementary Note: Residuals and Negatives

Figure S16 shows negatives (blue) in the solution for a range of depths. Both DSW and LSW negatives occur away from the continent and are generally higher in magnitude at shallower depths. Negatives are particularly high in the Pacific sector of the Southern Ocean between 60-120°W and either side of the 180°E meridian. Figure S17 plots the residuals of the solution for the same range of depths. The largest residuals, both positive and negative, occur in the same regions and also generally decrease as a function of depth. We interpret these areas as regions in which the water properties are out of range of the selected end members, suggesting that there are un-modelled water masses here. This is to be expected, given that our SWTs were selected to constrain the known near-shelf water mass configuration. Specifically, we choose just one CDW end member to constrain all sub-mixed layer waters away from the shelf. This is a reasonable approximation near the shelf, where CDW upwells from depth, but it breaks down further north where mode and intermediate waters are formed. Given that our analysis is focused on the shelf and near-shelf conditions, where negatives and residuals are small, we do not consider these issues of importance to our conclusions.

S9 Supplementary Note: Sample Water Mass Classification Outputs

Figure S1 shows sample output from the classification for all of the SWTs in the analysis at a range of depths and longitudes.

S9.1 Supplementary Note: The cold fresh tail: ISW, WW or surface water?

As discussed in Section S1.2, we select a SWT to constrain the cold fresh tail of the T-S parameter space and label it as ISW. As is shown in Figure S1, this end member also encompasses a significant amount of surface and sub-surface waters. Given that our analysis is focused on near-shelf processes beneath the mixed layer, we do not consider this of great importance. Where we have high concentrations of fresh, cold water near the shelf at depth, we assume this to be ISW. This is supported by the latitude-longitude plots in Figure S1; ISW away from the shelf decreases with depth. A patch of ISW remains at 400 m and even 800 m in the Pacific sector, and we attribute this to the presence of cold and fresh mode or intermediate waters in this region. We also do not regard these waters as being important to near-shelf dynamics.

However, we do acknowledge that some of what we classify as ISW (i.e. the cold, fresh waters which do occur at depth near the shelf in Figure S1) is actually some of the capped fresh surface water known as Winter Water. We do not consider the distinction between these as fundamental to our analysis or conclusions.

Standard Error of MEOP Data				
Shelf Region	Mean θ ($^{\circ}C$)	θ Std. Error ($^{\circ}C$)	Mean S (psu)	S Std. Error (psu)
Weddell Sea	-1.84	0.0086	34.48	0.0035
Princess Martha Coast	-1.64	0.0086	34.18	0.0038
Cape Darnley	-1.20	0.0081	34.25	0.0037
Western Prydz Bay	-1.59	0.0079	34.25	0.0037
Eastern Prydz Bay	-1.38	0.0069	34.28	0.0036
Leopold/Astrid Coast	-1.80	0.0114	34.36	0.0051
Knox Coast	-0.87	0.0077	34.35	0.0035
Adelie Coast	-1.71	0.0061	34.37	0.0027
Ross Sea	-1.55	0.0027	34.38	0.0012
Amundsen Sea	-1.25	0.0048	34.17	0.0019
Bellingshausen Sea	-0.28	0.0042	34.16	0.0019

Table S1: Standard errors of each MEOP shelf region used in Section S8. Standard error is calculated as $\sigma/\sqrt{n}$, where σ is the standard deviation of the MEOP data at all the shelves, and n is the sample size at each shelf.

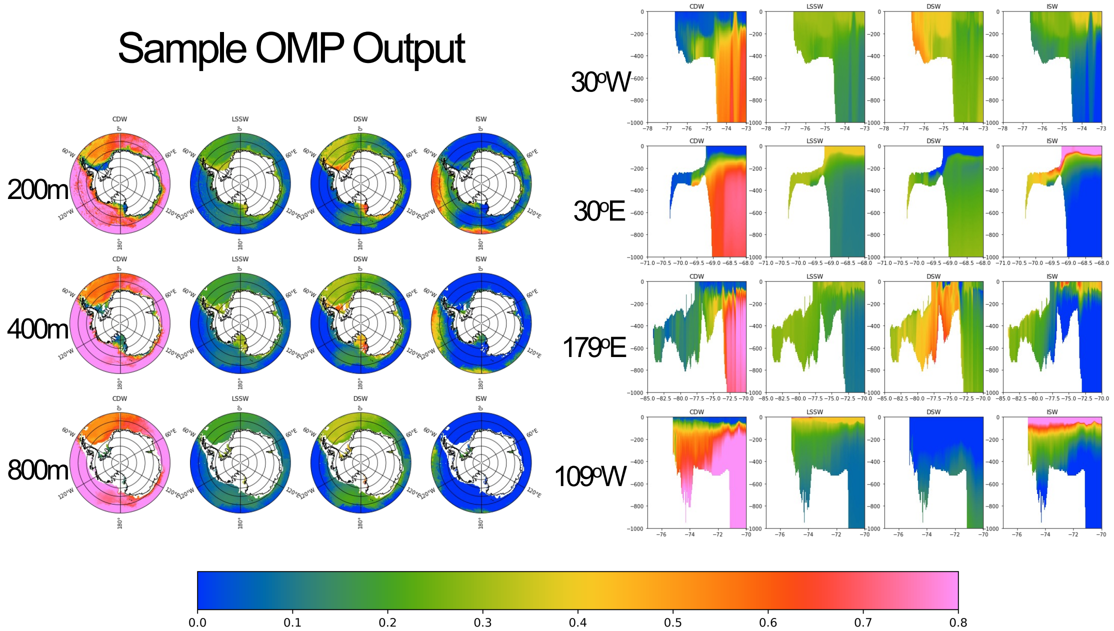

Figure S1: Sample water mass classification output. All four water masses defined in this study are shown here over a range of depths and longitudes on September 1st 2005.

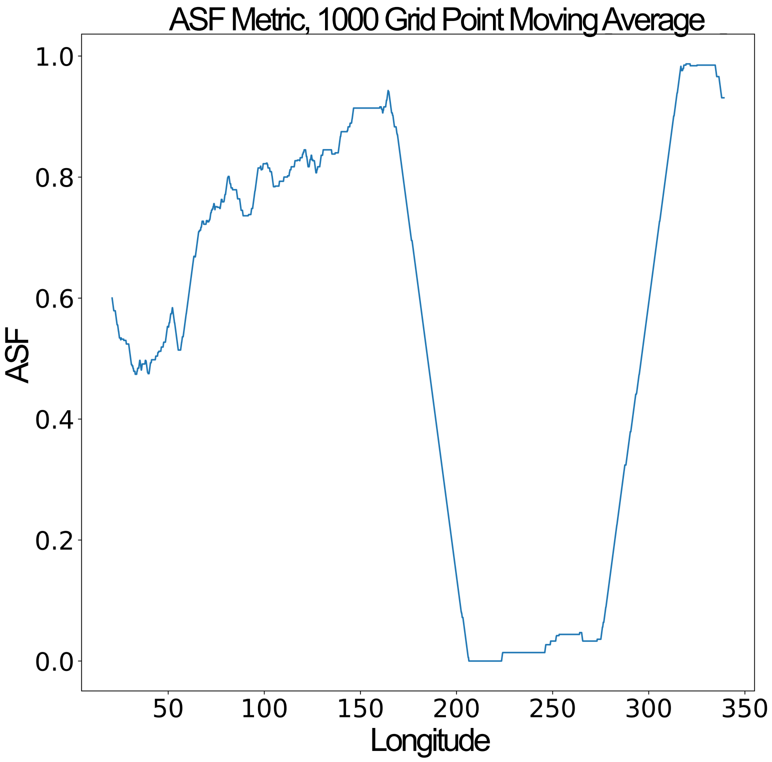

Figure S2: The Antarctic slope front metric. The ASF metric, defined in Section S1.1, is plotted here as a function of longitude with a 1000 grid point moving average.

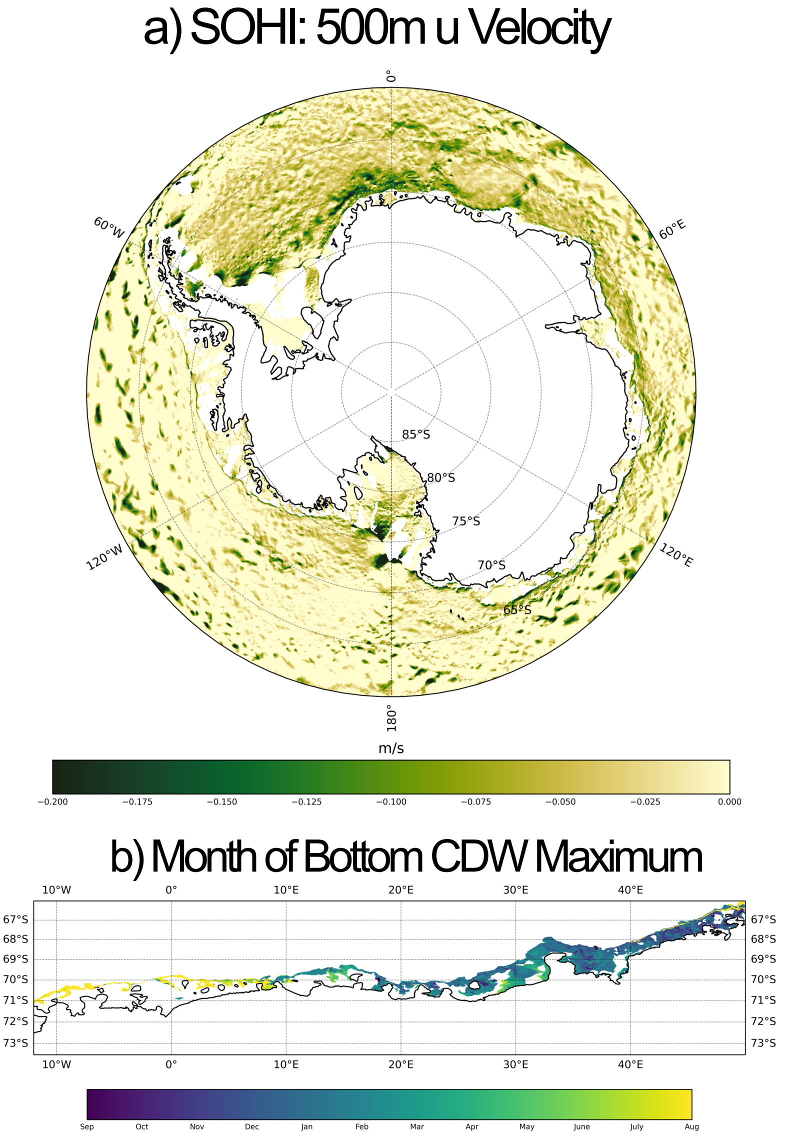

Figure S3: Advection of CDW in the far eastern Weddell Sea. Panel a) shows SOHI zonal velocity at 500 m for September 1st 2005, whilst b) plots the month at which the bottom maximum of CDW occurs, filtered by the seasonal variance as in Figure 4. Note that panel b) uses 12 24-hour averages on the first of each month.

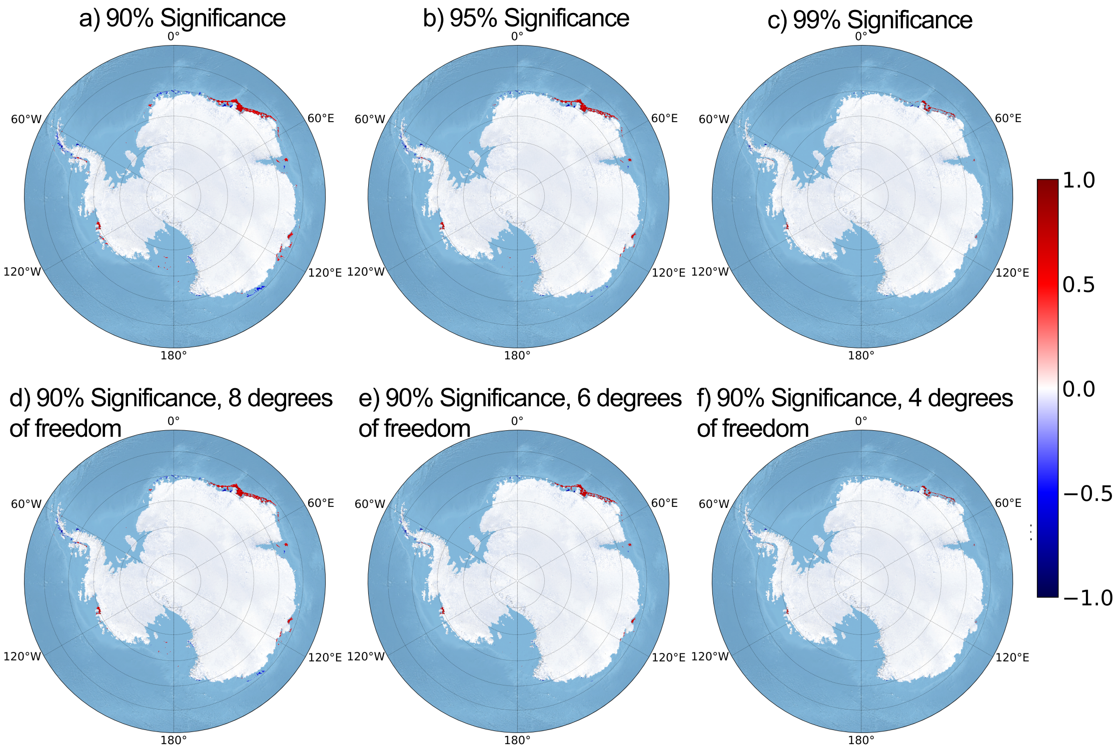

Figure S4: Significance of wind correlations. As for Figure 4b, but with all correlations which are not significant at a) 90, b) 95 and c) 99% removed. Panels d), e) and f) show the significance of the wind-CDW correlations at the 90% level, assuming 8, 6, and 4 degrees of freedom respectively.

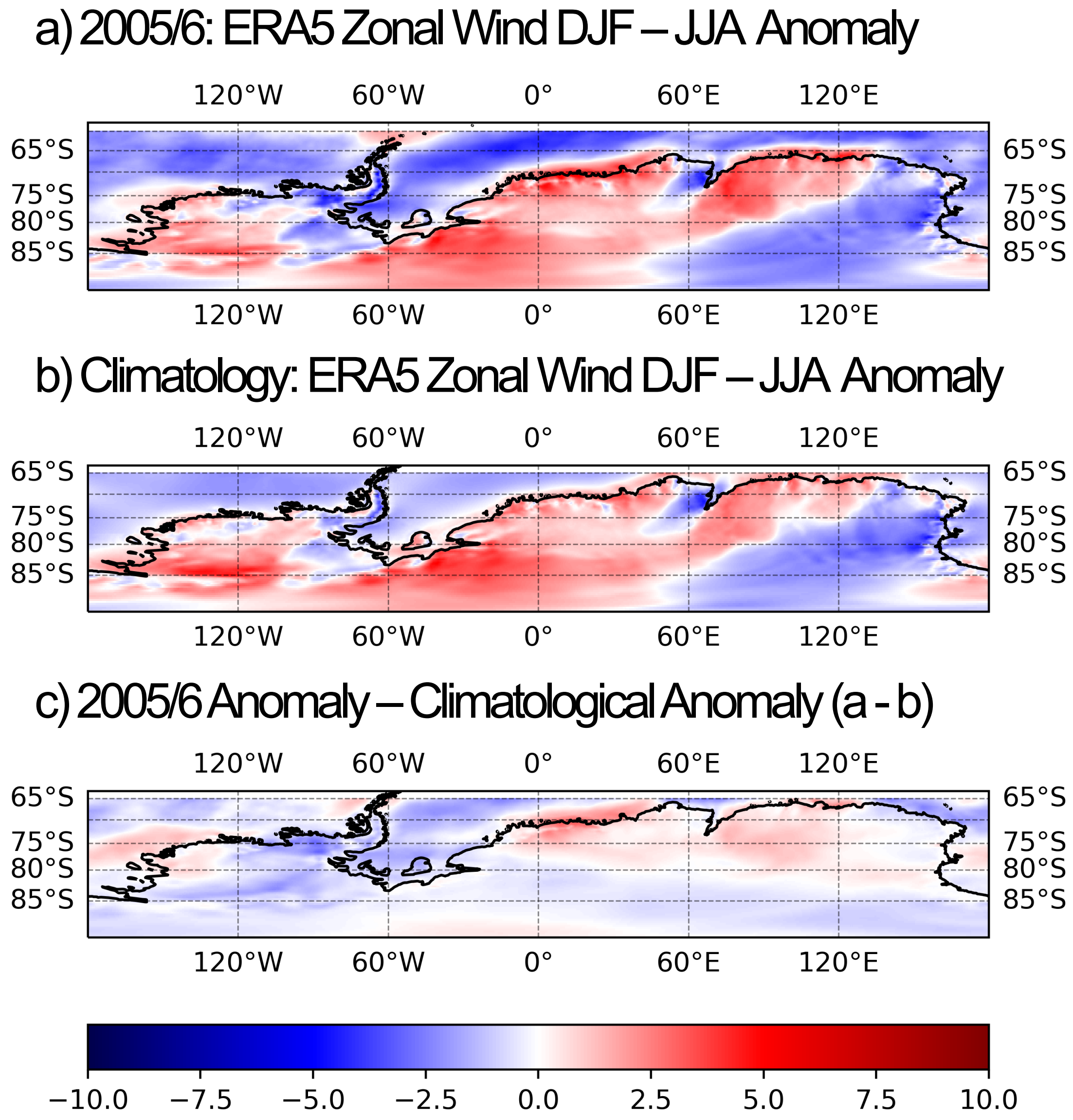

Figure S5: Seasonality in the ERA5 zonal winds. Panel a) shows the zonal wind stress summer (DJF) minus winter (JJA) anomaly composite for 2005/6. Panel b) shows the same, but for a climatological average (1980-2018). Panel c) takes the anomaly of a) and b), to show how the 2005/6 pattern of wind forcing differed from the long-term mean conditions.

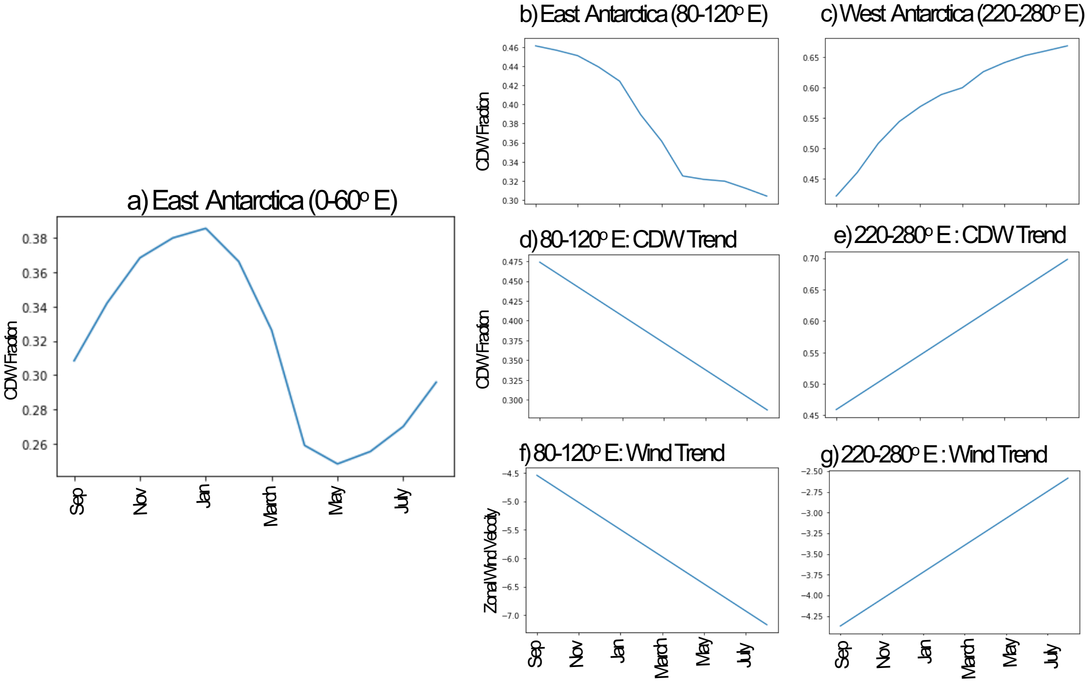

Figure S6: Time series of CDW fraction on the shelf at a) 0-60°E, b) 0-60°E, and c) 0-60°E, alongside the linear trends in these latter two time series, shown in panels d and e. Panels f and g show the linear trend in the ERA5 zonal surface wind time series for the same locations. Only the points shown in Figure 4 (i.e. high CDW variability locations) are used in the calculation.

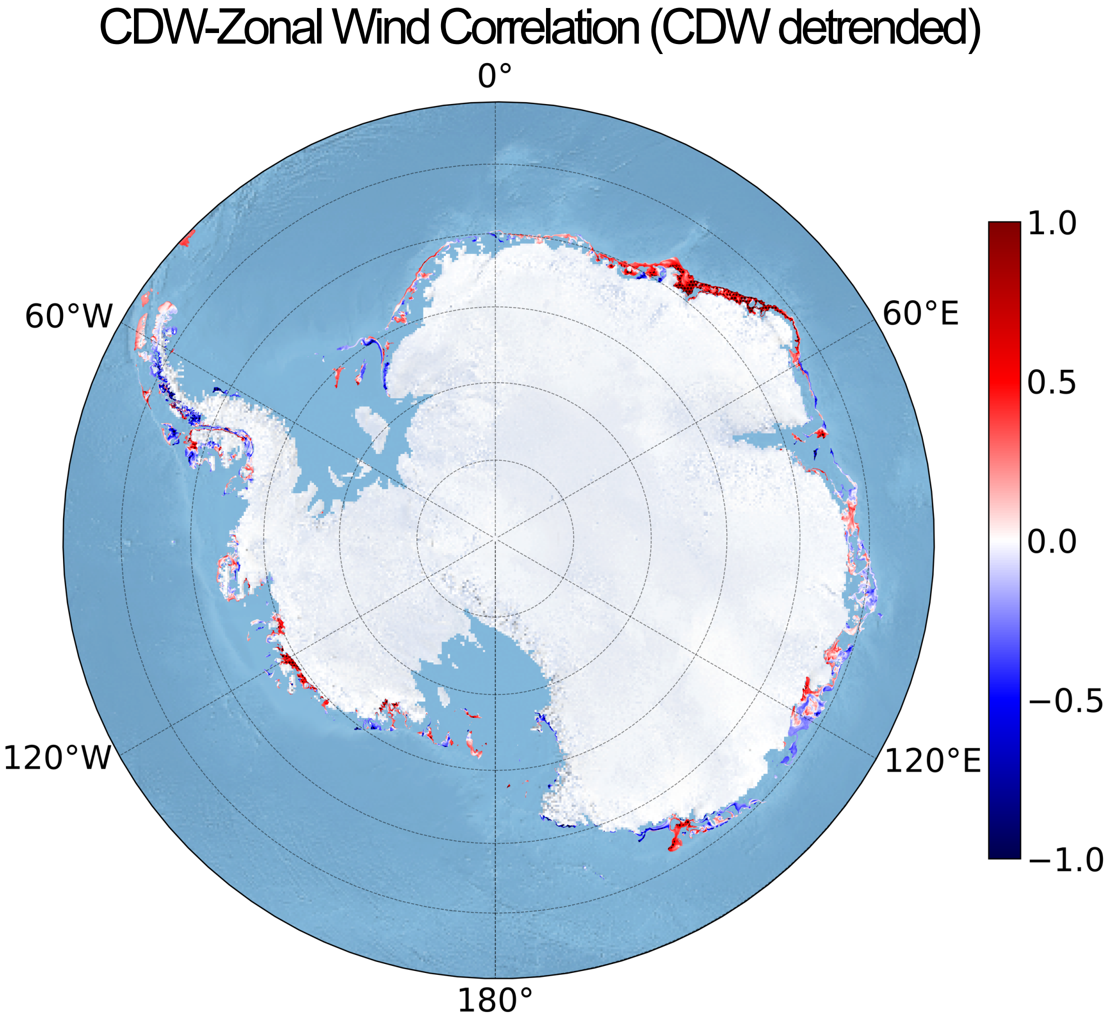

Figure S7: As for Figure 4b, but at each location the CDW time series is detrended.

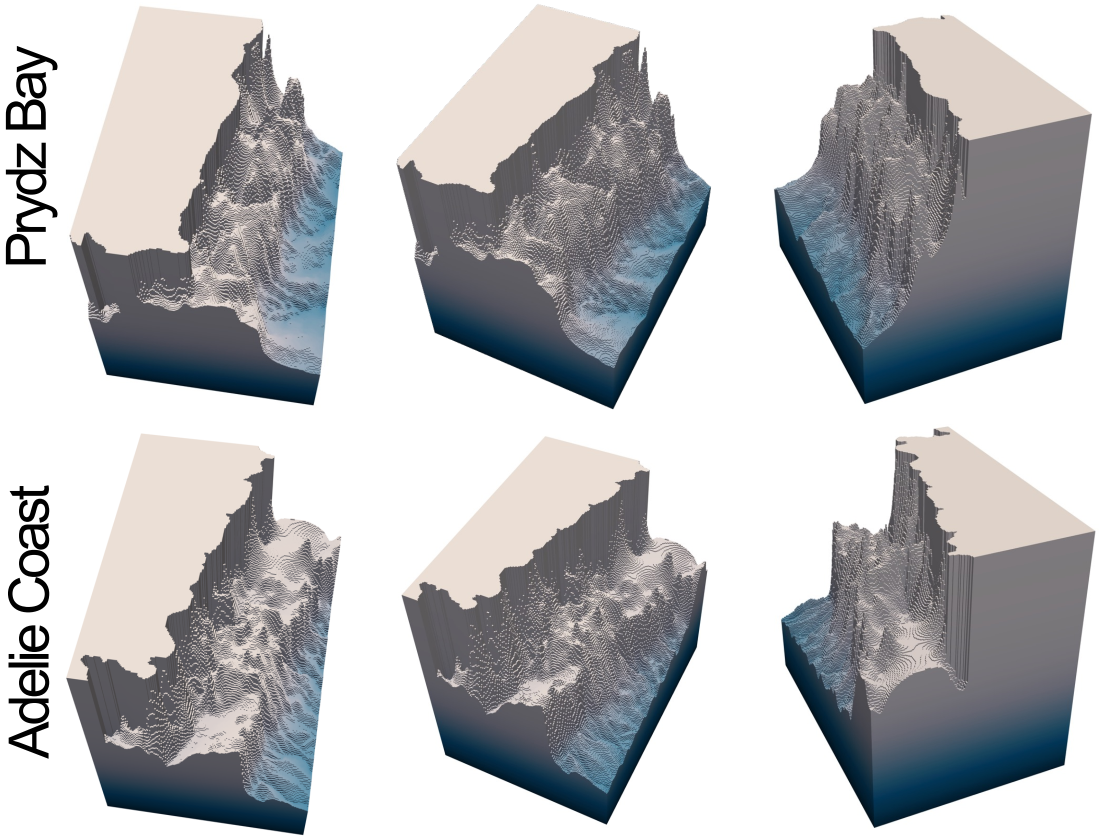

Figure S8: Additional angles of the 3-D bathymetry of Prydz Bay and the Adélie Coast.

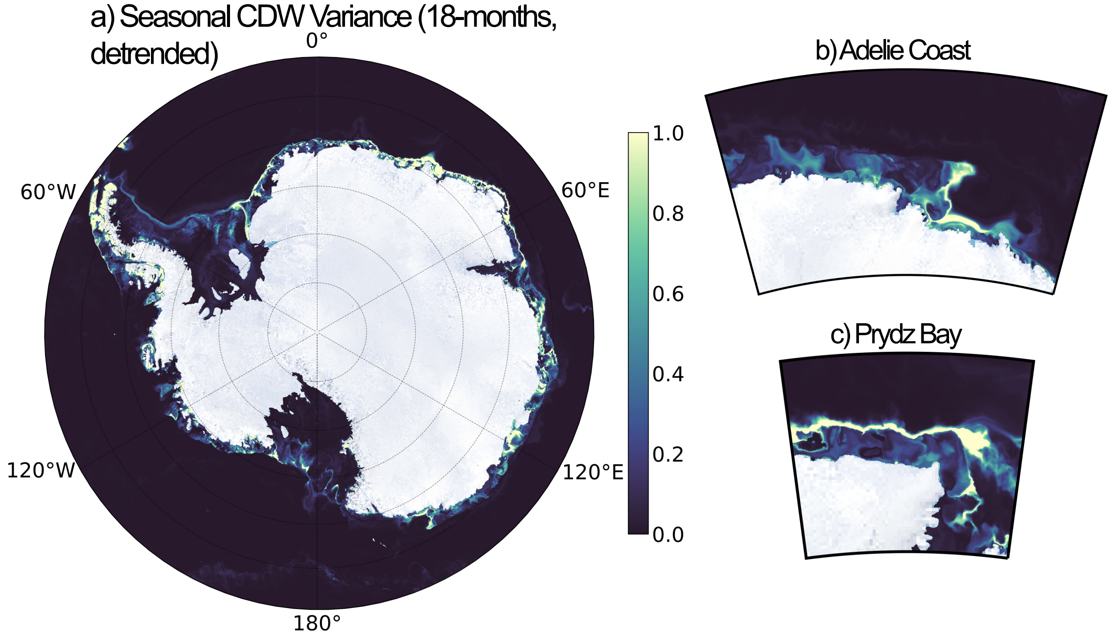

Figure S9: a) As for Figure 3b, but with the CDW time series extended to 18-months and detrended. Panels b) and c) show close ups of panel a), for the Adelie Coast and Prydz Bay respectively.

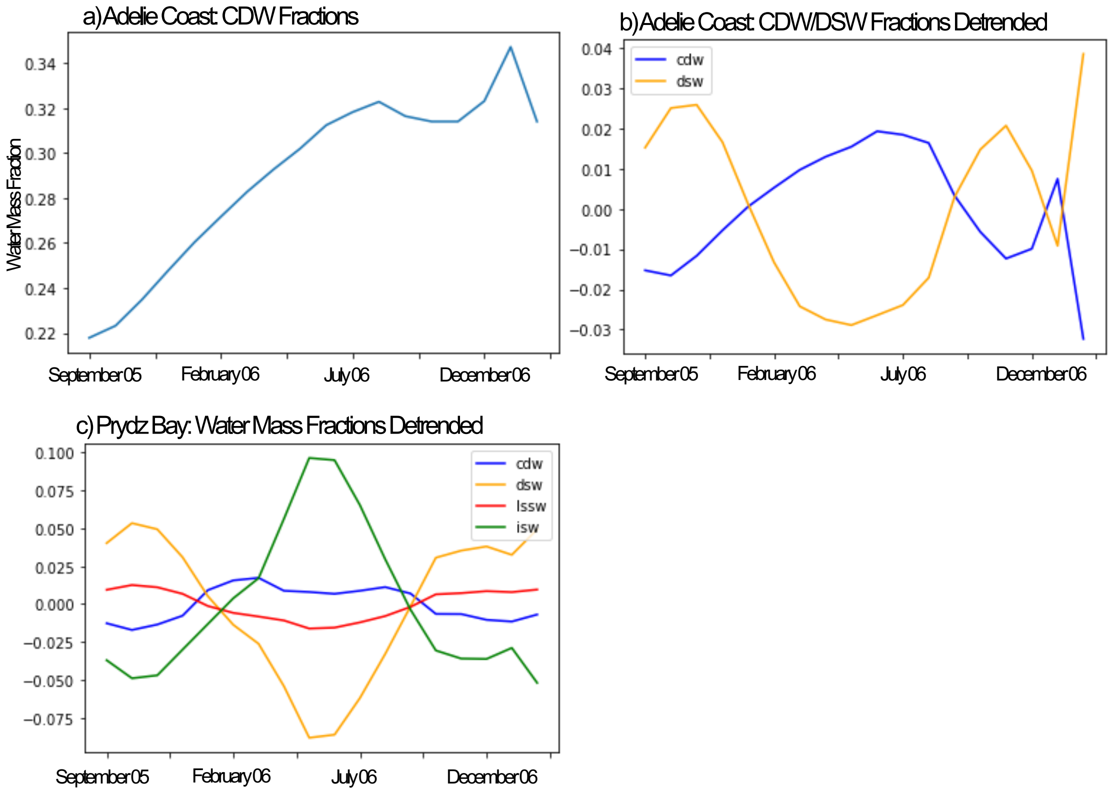

Figure S10: Panel a) shows the same CDW time series as in Figure 8c, but extended to an 18-month duration. Panel b) shows the time series in panel a) but de-trended, alongside the equivalent time series for DSW. Panel c) shows the same as panel b), but for Prydz Bay (i.e. the extended and de-trended time series of Figure 9c) . The equivalent time series for LSSW and ISSW are also added to panel c).

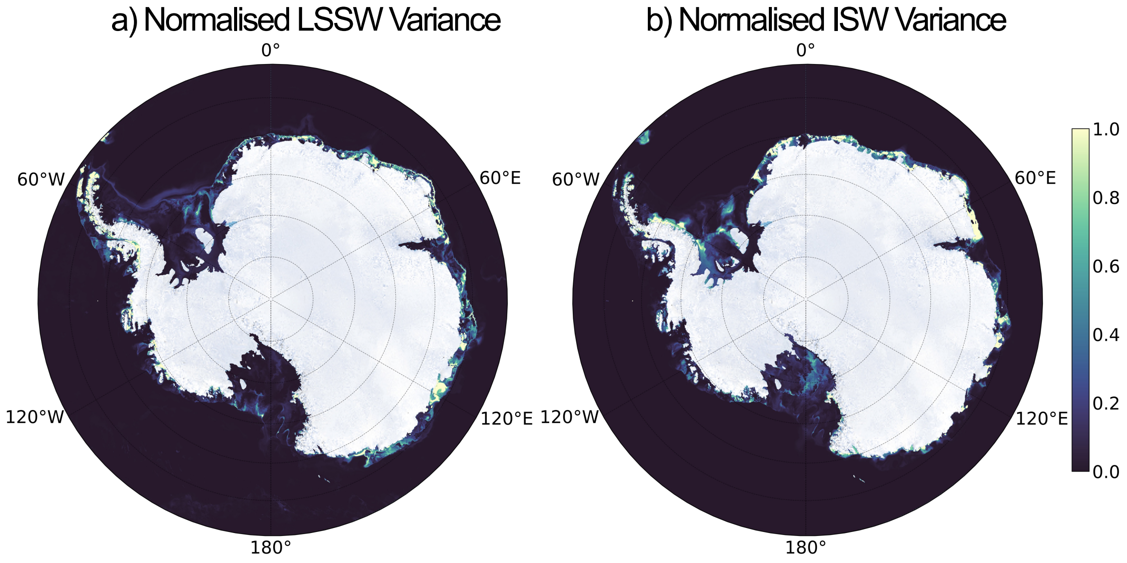

Figure S11: As for Figure 3b, but showing the seasonal variance for a) LSSW and b) ISW instead of CDW.

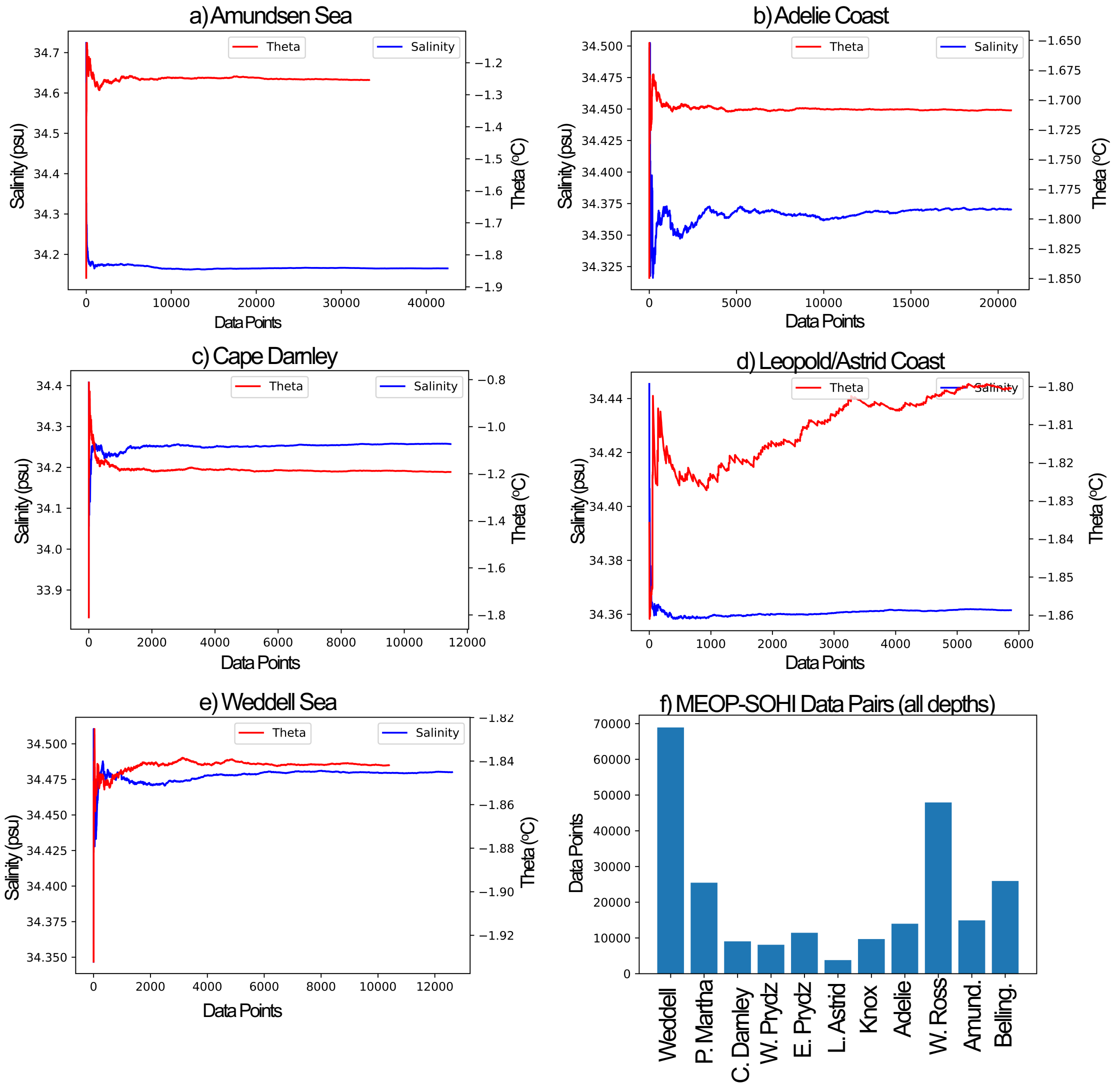

Figure S12: Convergence sampling in MEOP seal data. The number of MEOP data points needed to reach temperature and salinity convergence is shown for a range of sample regions. MEOP data are randomly sampled from the near-shelf area and used to calculate a new mean with n+1 data points, which is plotted in red for temperature and blue for salinity. The total number of data points (MEOP-SOHI pairs) in each region is shown at the bottom right.

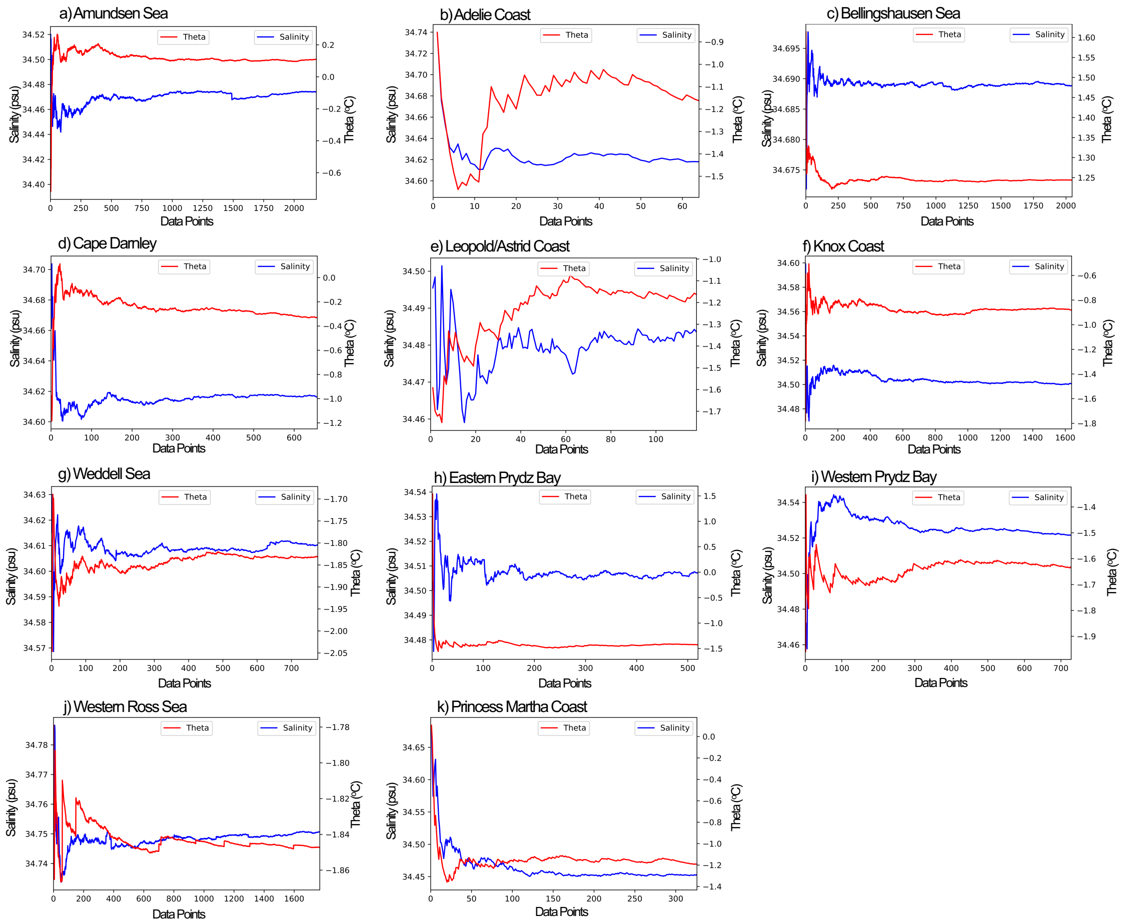

Figure S13: Convergence sampling in MEOP seal data below 400m for all regions. MEOP data are randomly sampled from the near-shelf area and used to calculate a new mean with n+1 data points, which is plotted in red for temperature and blue for salinity.

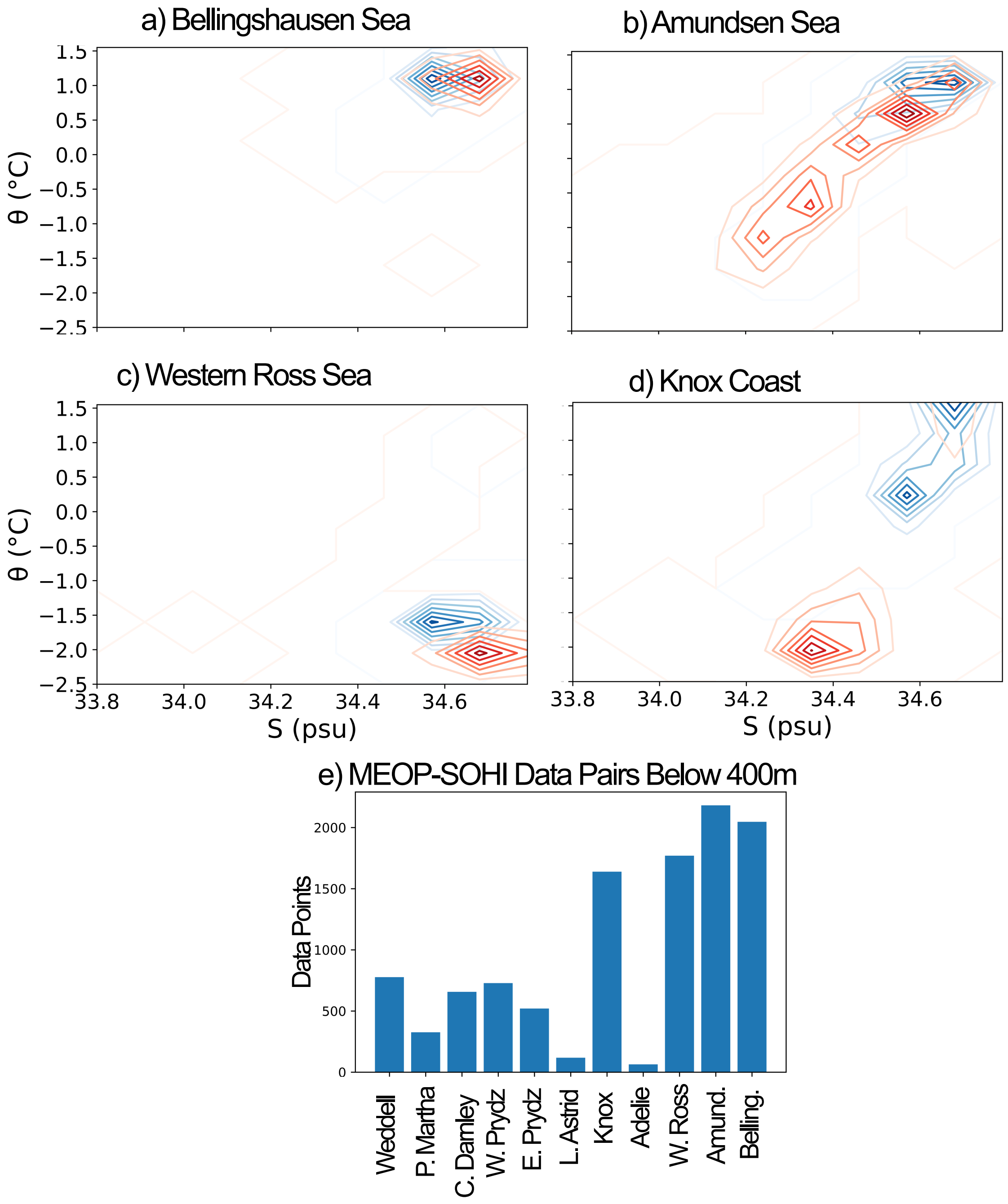

Figure S14: Depth decomposition of the four most sampled regions below 400 m: the Bellingshausen sea, Amundsen sea, Western Ross sea, and Knox Coast. Panels a), b), c), and d) are the same as the bivariate histograms in Figure 2, but any measurements above 400 m are removed. Panel e) shows the MEOP data density below 400 m in each region.

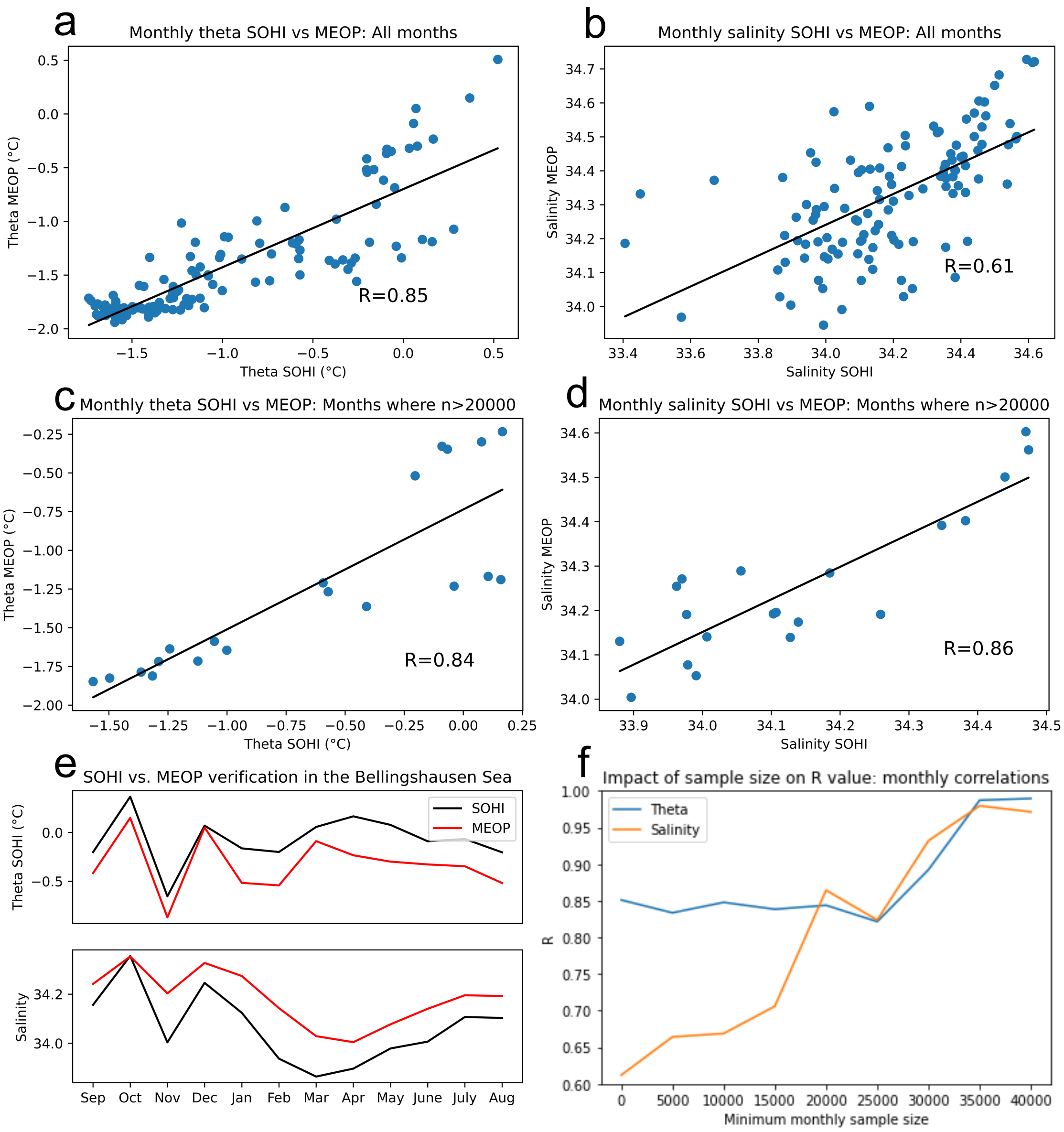

Figure S15: Verification of the SOHI seasonal cycle with MEOP data. Figures a) and b) plot monthly temperature and salinity of SOHI against MEOP data. Each data point is a monthly-mean at a given near-shelf region (see Figure 2). Figures c) and d) are the same, but this time only include monthly means where the data coverage exceeds 20,0000 data points. Figure e) shows a sample seasonal comparison for the Bellingshausen Sea. Figure f) shows the impact of increasing the minimum monthly sample size on the SOHI-MEOP r value (such as those in a, b, c and d) for both temperature and salinity.

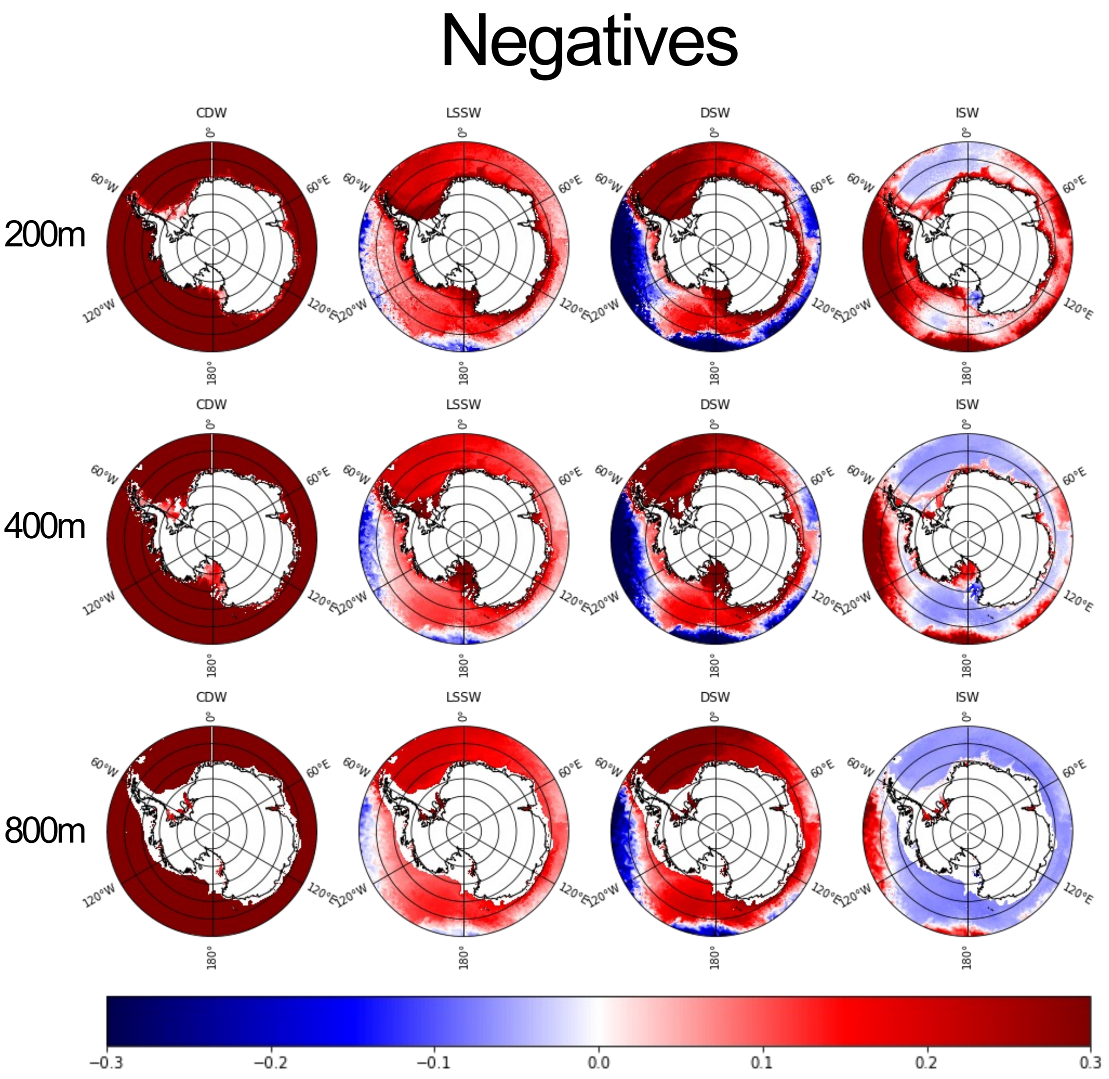

Figure S16: Negatives in the water mass classification solution. The solution for all four water masses at a range of depths on September 1st 2005, with a colorbar adjusted to show negatives.

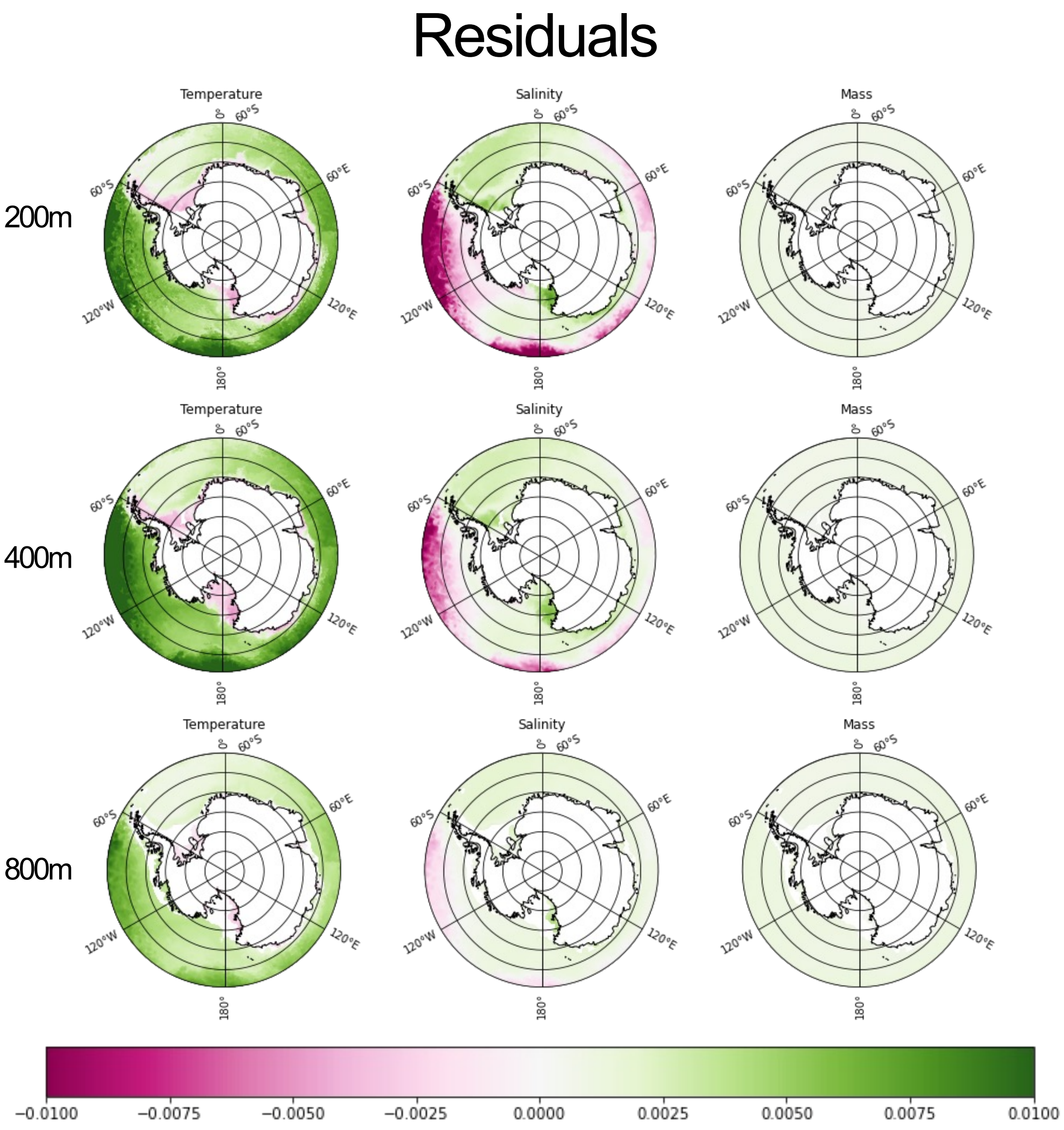

Figure S17: Residuals in the water mass classification solution. Residuals for all three variables in the solution at a range of depths on September 1st 2005.

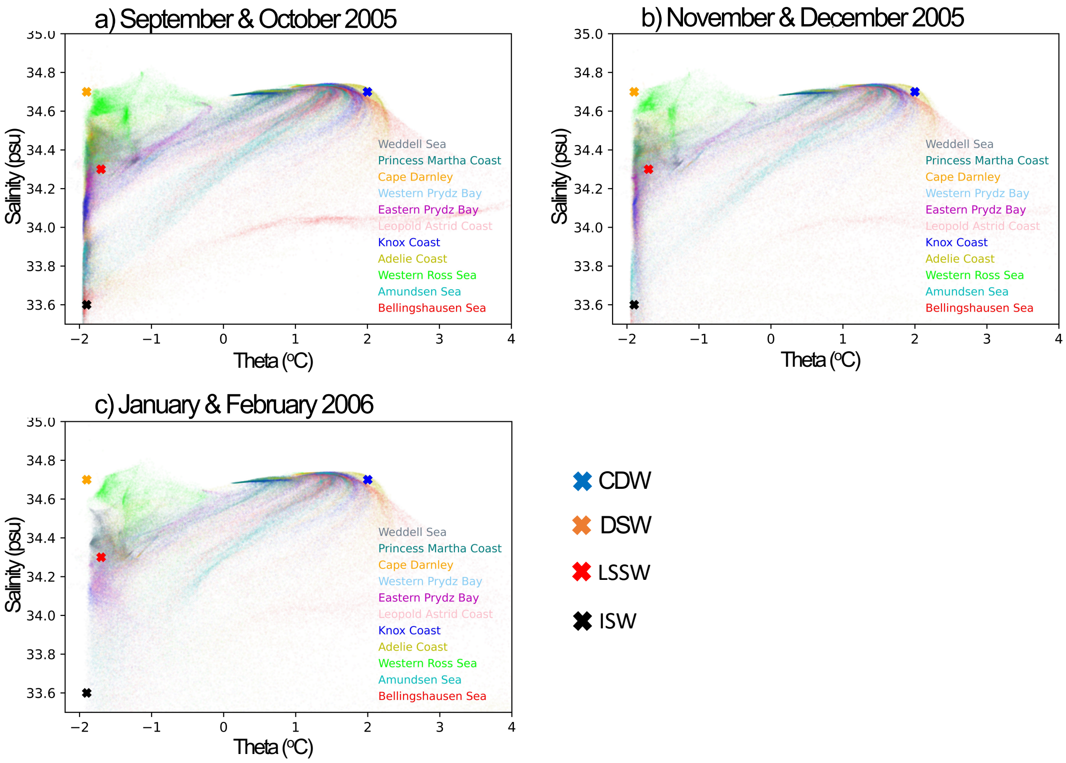

Figure S18: Water mass end members used in this study. Temperature-salinity distributions for a range of months, showing the four end members used in this study. Data points are sampled from the regions in Figure 2 and coloured accordingly.

Supplementary References

1. Tomczak, M. A multi-parameter extension of temperature/salinity diagram techniques for the analysis of non-isopycnal mixing. en. *Progress in Oceanography* **10,** 147–171 (Jan. 1981).
2. Schodlok, M. P. *et al.* Ice shelf basal melt rates around Antarctica from simulations and observations. *Journal of Geophysical Research: Oceans* **121,** 1085–1109 (Feb. 2016).
3. Williams, G. D. *et al.* The suppression of Antarctic Bottom Water formation by melting ice shelves in Prydz Bay. en. *Nature Communications* **7,** 12577 (Aug. 2016).
4. Dawson, H. R. S. *et al.* Pathways and timescales of connectivity around the Antarctic continental shelf. en. *Journal of Geophysical Research: Oceans* **128,** e2022JC018962 (Feb. 2023).
5. Moffat, C. & Meredith, M. Shelf–ocean exchange and hydrography west of the Antarctic Peninsula: a review. en. *Philosophical Transactions of the Royal Society A: Mathematical, Physical and Engineering Sciences* **376,** 20170164 (June 2018).
6. Moffat, C. *et al.* On the characteristics of Circumpolar Deep Water intrusions to the west Antarctic Peninsula Continental Shelf. en. *Journal of Geophysical Research: Oceans* **114,** 2008JC004955 (May 2009).
7. Martinson, D. G. & McKee, D. C. Transport of warm upper circumpolar deep water onto the Western Antarctic Peninsula Continental Shelf. *Ocean Science* **8,** 433–442 (2012).
8. Treasure, A. *et al.* Marine Mammals Exploring the Oceans Pole to Pole: A Review of the MEOP Consortium. *Oceanography* **30,** 132–138 (June 2017).
9. Narayanan, A. *et al.* Water mass characteristics of the Antarctic Margins and the production and seasonality of Dense Shelf Water. *Journal of Geophysical Research: Oceans* **124,** 9277–9294 (Dec. 2019).
10. McMahon, C. R. *et al.* Animal Borne Ocean Sensors – AniBOS – An Essential Component of the Global Ocean Observing System. en. *Frontiers in Marine Science* **8,** 751840 (Nov. 2021).
